# Local structure and electrochemical performances of sulfurized polyethylene glycol after heat treatment

**DOI:** 10.1038/s41598-020-74118-5

**Published:** 2020-10-09

**Authors:** Nobuhiko Takeichi, Toshikatsu Kojima, Hiroshi Senoh, Hisanori Ando

**Affiliations:** Research Institute of Electrochemical Energy (RIECEN), National Institute of Advanced Industrial Science and Technology (AIST), 1-8-31 Midorigaoka, Ikeda, Osaka 563-8577 Japan

**Keywords:** Batteries, Batteries

## Abstract

Designing a high-capacity positive electrode material is critical for the advancement of lithium-ion batteries. Sulfurized polyethylene glycol (SPEG), containing ca. 61 wt% of sulfur, is a promising positive electrode material that exhibits a large initial discharge capacity of more than 800 mAh g^−1^. In this study, we present the local structure and electrochemical performances of SPEG. A high-energy X-ray total scattering experiment revealed that sulfur in SPEG is predominantly fragmented and bound to carbon atoms. The changes in the physicochemical properties of SPEG due to heat treatment with nitrogen gas at various temperatures were investigated using thermogravimetric analysis, Raman spectroscopy, X-ray absorption near edge structure, and extended X-ray absorption fine structure. Comparing the electrochemical performances of SPEG after heat treatment at various temperatures, it was found that S–S and C=S bonds contribute to the overall electrochemical performance of SPEG.

## Introduction

Rechargeable lithium-ion (Li-ion) batteries are widely used as electric power sources in electric vehicles, mobile phones, and power backup systems because of their high energy densities. Conventional Li-ion batteries contain several minor metals, such as nickel, cobalt, and manganese, which may cause serious resource depletion. Recently, sulfur is attracting attention as an active material for Li-ion batteries. Considering industrial requirements, sulfur is cost-effective and environmentally friendly^1^. In addition, sulfur is a two-electron transfer material with a theoretical capacity of 1672 mAh g^−1^. Therefore, lithium–sulfur (Li–S) battery is an interesting topic of research with respect to next-generation energy-storage devices^2^. The durability (or cycle performance) of secondary batteries is also an important consideration for practical applications^3^. The positive electrode active material, sulfur, has some disadvantages such as high electric resistance and high solubility^4^. Accordingly, the dissolution of the positive electrode active material leads to severe battery degradation.

To suppress the dissolution of sulfur, several approaches, such as physical inclusion^5–8^, chemical fixation^9,10^, and sulfur-containing polymers^11,12^, have been investigated thus far. Nevertheless, no breakthrough technology leading to practical use has been reported. Watanabe and others suggested a new electrolyte solution called “solvate ionic liquid” that comprised a 1:1 molar mixture of lithium bis(trifluoromethylsulfonyl)amide (LiTFSA) and triglyme (G3) or tetraglyme (G4)^13^. There are few free solvents in the mixture that suppress the dissolution of sulfur.

In our previous study, we proposed a novel sulfur–carbon composite prepared by the integration of sulfur and polyethylene glycol (SPEG)^14^. SPEG shows a large initial discharge capacity of ca. 800 mAh g^−1^ in a half cell with lithium metal. The characterization of SPEG was carried out to clarify the structure. As we previously reported, no characteristic X-ray powder diffraction (XRD) pattern was recorded for sulfur. According to Raman spectroscopy, the characteristic peak at 1441 cm^−1^ (assignable to the C–S bond) was observed to emerge^15^; however, the intrinsic peaks at 1330 cm^−1^ and 1561 cm^−1^ did not appear, which can be attributed to the D- and G-bands of graphite carbon, respectively^16^. In addition, a weak peak is observed at 481 cm^−1^, which lies in the fundamental vibration region of pure sulfur. These results indicate the presence of fragmented sulfur and its strong bond with the carbon atom. Based on these characterizations, it can be concluded that the SPEG structure is significantly different from other sulfurized carbonaceous electrode materials^9^.

In this study, we first investigated the thermal stability of SPEG after the heat treatment at various temperatures to understand the local structure of SPEG in detail. Subsequently, the electrochemical performances were analyzed in a half cell wherein LiTFSA/G4 solvate ionic liquid was used as an electrolyte. Further, the analysis of the local structure of SPEG was performed via high-energy X-ray total scattering, and the sulfur content and phase changes were determined. Comparing the results of the X-ray absorption near-edge structure (XANES) and extended X-ray absorption fine structure (EXAFS), the relationship between the local structure of SPEG and its different electrochemical performances was discussed.

## Results and discussion

### Physicochemical properties of SPEG after the heat treatment

**Figure 1:**
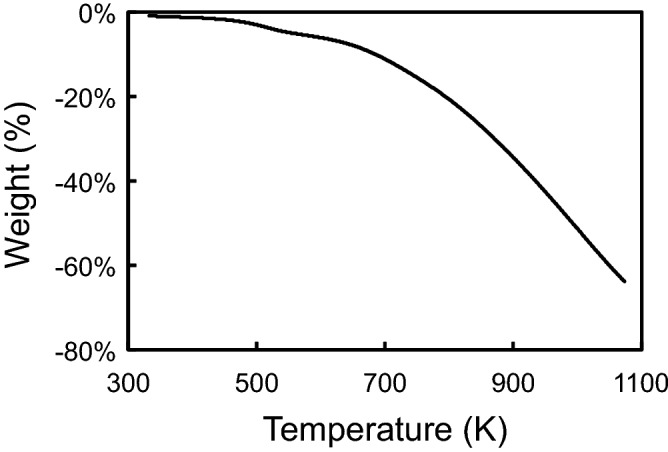
Thermogravimetric profile of SPEG at the heating rate of 20 K min^−1^ under an argon atmosphere.

**Figure 2:**
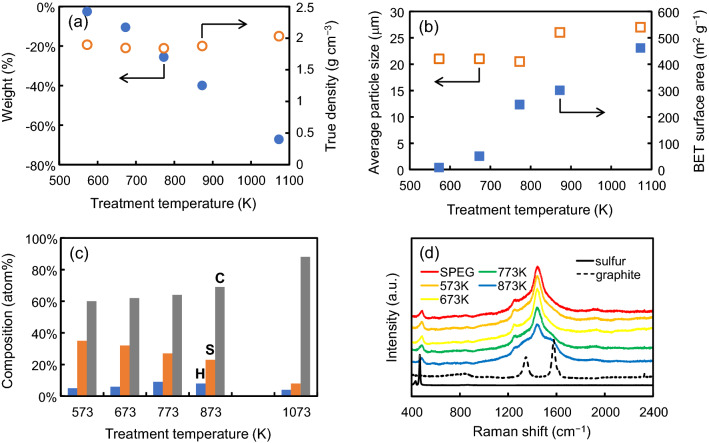
Physicochemical properties of SPEG after heat treatment at different temperatures: (**a**) weight loss and true density, (**b**) average particle size and BET surface area, (**c**) chemical composition, and (**d**) Raman spectra.

Figure 1 shows the thermogravimetric (TG) profile of SPEG under an argon atmosphere at the rate of 20 K min^−1^. No drastic weight loss was observed even at temperatures higher than 718 K, which is the boiling point of sulfur. This indicates that sulfur is tightly bound to the carbon matrix. The weight loss and true density of SPEG after the heat treatment are shown in Fig. 2a. The weight of SPEG hardly changed by the heat treatment at 573 K for 1 h, indicating that desulfurization during sample preparation prior to the heat treatment is sufficient. An increase in temperature decreases the weight of SPEG, and only one third of SPEG remains after 1073 K. This result is almost consistent with that of the TG analysis (Fig. 1). The true density of SPEG did not change up to 873 K. Figure 2b shows the dependence of temperature on the particle size and surface area of SPEG. Above 873 K, the average particle size of SPEG was slightly larger than that below 773 K, while the surface area was significantly larger. This means that the surface of SPEG turns rough after the heat treatment at higher temperatures, or alternatively, micro pores are formed. As shown in Fig. 2c, the atomic ratio of sulfur in SPEG decreases with increase in temperature. This clearly indicates that SPEG underwent thermal decomposition and sulfur was released from the SPEG structure. Above 773 K, the sulfur content reduces to less than half of carbon, which affects the electrochemical performance of SPEG. Figure 2d shows the change in Raman spectra of SPEG with respect to the heat treatment. The main peak at 1441 cm^−1^, which appeared in SPEG, weakened with an increase in temperature, while two peaks near D-band (1330 cm^−1^) and G-band (1561 cm^−1^) which originated from the graphite structure, strengthened. The decrease in sulfur content, as shown in Fig. 2c, is probably related to the disappearance of the main peak. Fourier-transform infrared (FT-IR) spectroscopy was carried out for tracing the changes in the sub-surface characteristics of SPEGs by the heat treatment. Compared to the profile of standard compounds (rubeanic acid and L-cystine), a weak peak at 1240 cm^−1^, assigned to C=S bond, was observed in the samples treated below 873 K. No characteristic peak assigned to the C–S bond was found in any of the SPEG samples (Supplementary Figure S1).

**Figure 3:**
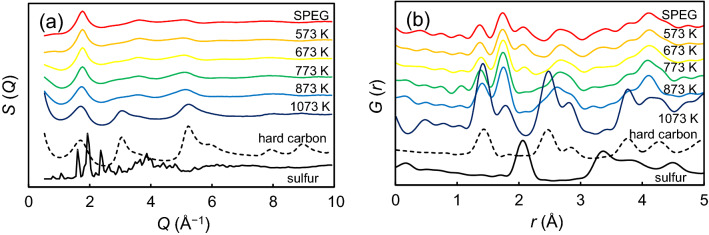
Local structures of SPEG after heat treatment at different temperatures: (**a**) structure factors, and (**b**) total pair correlation functions.

X-ray total scattering is frequently utilized to elucidate the local structure of amorphous materials that cannot be classified by using XRD measurements^17^. To investigate the local structure of SPEG in detail, X-ray total scattering measurements were obtained. Figure 3a shows the structure factor, *S*(*Q*), of SPEG before and after heat treatment at different temperatures. For reference, the *S*(*Q*) values of sulfur and hard non-graphitic carbon are also shown in this figure. The temperature profiles up to 873 K were almost the same, exhibiting the high stability of the SPEG structure. In contrast, small peaks at 3.1 and 5.3 Å^−1^ appeared above 1073 K, which were similar to those of hard carbon. This indicates that the heat treatment turns the SPEG into a hard carbon-like structure with a large surface area. The total pair correlation function, *G*(*r*), obtained from the Fourier transformation of *S*(*Q*) is shown in Fig. 3b. SPEG showed an intensive peak at 1.8 Å, which can be assigned to the C–S bond because the lengths of the C–S bonds for methanethiol^18^ and thiophene^19^ are 1.82 and 1.74 Å, respectively. It is worth noting that the peak appearing at 2.1 Å (assignable to the S–S bond) is rather small. This indicates that sulfur is mostly fragmented in SPEG. The peak cannot be observed above 773 K, thereby indicating that the S–S bond disappears in SPEG. In addition, a peak appeared at 1.4 Å, which was also observed in SPEG, corresponding to the distance between the C–C bond in hard non-graphitic carbon. It can be summarized that SPEG has a hard carbon framework structure that is stable up to 873 K and contains the C–S bond in its local structure. However, further investigation is necessary to clarify the details.

**Figure 4:**
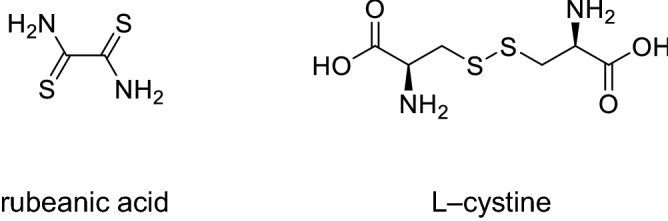
Chemical structures of rubeanic acid (left) and L-cystine (right).

**Figure 5:**
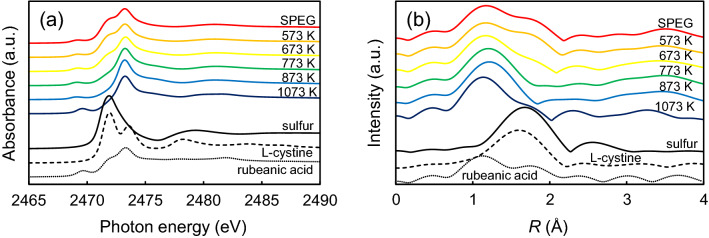
Local structures of SPEG after heat treatment at different temperatures: (**a**) S *K*-edge XANES spectra and (**b**) S *K*-edge EXAFS spectra.

In this study, we focused on the bond characteristics of carbon and sulfur. As shown in Fig. 4, rubeanic acid and L-cystine were selected as the model compounds. Rubeanic acid comprises two thioketone groups (C=S) in its molecular structure. L-cystine seems to be a suitable compound that contains both S–S and C–S bonds. Figure 5a shows the S *K*-edge XANES spectra of SPEG, before and after heat treatment, which are apparently different from those of sulfur. Four specific peaks, 2469, 2472, 2473, and 2482 eV, are similar to those of the rubeanic acid, rather than those of the L-cystine. Thus, it is speculated that the structure of SPEG exhibits a C=S bond character (not C–S bond). With the increase in temperature, the peak at 2472 eV, which is a characteristic peak of sulfur, disappeared gradually; meanwhile, the other three peaks hardly changed. S *K*-edge EXAFS spectra also support the hypothesis (Fig. 5b). The spectrum of SPEG was similar to that of rubeanic acid. It is apparent that the peaks of the S–S bond in sulfur and L-cystine were observed in SPEG at 573 K and 673 K, respectively; these peaks disappeared above 773 K. However, peaks of the C=S bond, such as those of rubeanic acid, were observed even at 1073 K.

In this study, the XAFS data were collected with a bulk-sensitive total electron yield (TEY) detector. To obtain the surface information of SPEGs, X-ray photoelectron spectroscopy (XPS) was performed. The representative results are shown in Supplementary Figure S2. Before the heat treatment, a strong peak at 163.8 eV (assignable to the C–S bond) was observed for S(2p). A weak peak at 161.4 eV corresponding to the C=S bond was also observed as a shoulder, which faded for the samples above 873 K; this observation was consistent with the XAFS results. A small peak at 168.1 eV was observed even after a temperature of 1073 K was achieved; it is possible that some sulfur species interacted with oxygen and did not contribute to the electrochemical reactions.

### Electrochemical performances of SPEG after the heat treatment

**Figure 6:**
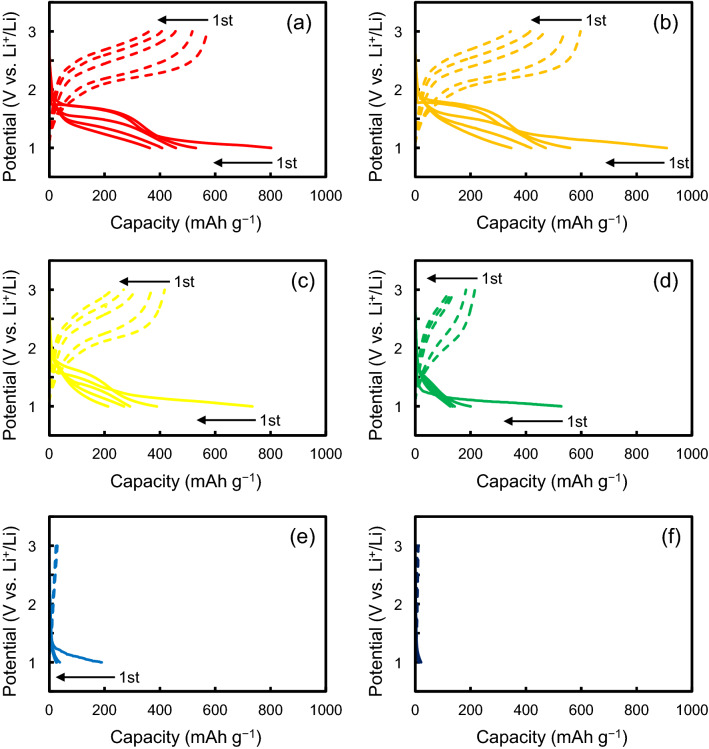
Charge/discharge profiles of SPEG in a half cell after heat treatment at different temperatures: (**a**) as prepared, (**b**) 573 K, (**c**) 673 K, (**d**) 773 K, (**e**) 873 K, and (**f**) 1073 K.

The electrochemical performances of SPEG in a half cell after the heat treatment at different temperatures are shown in Fig. 6. In the case of SPEG before the heat treatment (Fig. 6a), there are two plateaus during the first discharge, while only one plateau appears during the charge process. Table 1 shows a direct comparison of SPEG with respect to energy density against conventional cathodes. The average discharge potential of SPEG is approximately 1.8 V (vs. Li/Li^+^), while those of the conventional cathode materials such as LiCoO_2_ and LiFePO_4_ are higher than 3 V. However, the SPEG exhibits an initial discharge capacity of 800 mAh g^−1^, which is nearly 3 times the energy density of other materials. It is obvious that SPEG is one of the promising cathode materials.

**Table 1:** Energy density of conventional cathodes and SPEG.

Cathode	Energy density (Wh kg^−1^)	Ref.
LiCoO_2_	532	^24^
LiFePO_4_	495	^24^
LiNi_0.1_Mn_0.1_Co_0.8_O_2_	600	^25^
SPEG	1440	This work

**Figure 7:**
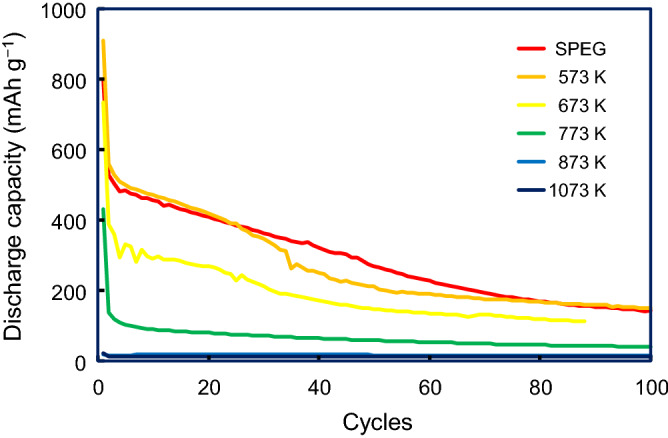
Cycle stability profiles of SPEG in a half cell after heat treatment at different temperatures.

A large irreversible capacity of approximately 250 mAh g^−1^ is observed in the subsequent potential–charge plots, which probably arises from the side reactions; for example, the firm formation on the electrode surface. The second discharge curve is slightly different from the first one, although both first and second charge curves appear to be similar. Moreover, a slight decrease in the voltage during discharge is ascribed to an increase in overpotential, probably induced by the resistance. With an increase in temperature during the heat treatment, the plateau regions in both the discharge/charge process became smaller. Only the first discharge could be observed after 873 K, and almost no electrochemical activity occurred above 1073 K, although sulfur remained in the sample (Fig. 2c). X-ray scattering and true density results reveal that the bulk structures above 1073 K are significantly different from the as-prepared SPEG. The cycle stability of SPEG after the heat treatment is presented in Fig. 7. It should be noted that the battery test began from the discharge process because SPEG has no lithium in the initial stage. The coulombic efficiency of each battery is shown in Supplementary Figure S3. In this study, the coulombic efficiency is defined as [*n*th charge capacity]/[*n*th discharge capacity]. It is clear that after several cycles, the coulombic efficiencies of the electrodes treated below 773 K are nearly 100%, while those above 873 K increased with increase in the cycle number. The discharge capacities of SPEG after the 573 and 673 K heat treatment exhibit gradual degradation, similar to that of the as-prepared SPEG (without the heat treatment), although the capacity after 100 cycles exceeds 100 mAh g^−1^. The capacity of SPEG after 773 K seems to be stable after 5 cycles. For long-term stability, sulfur dissolution is known to cause severe battery degradation in Li–S batteries^20^. To confirm sulfur dissolution, we analyzed the electrolyte solution after a battery test. However, sulfur dissolution from the cathode was less than 1%. This suggests that, in the SPEG system, the dissolution of sulfur in SPEG is not significant for degradation and that the amount of ‘non-active sulfur’ increases during charge/discharge cycling. One possible reason is the formation of the solid electrolyte interphase (SEI) on the electrode. The reason for capacity fading is still under investigation.

**Figure 8:**
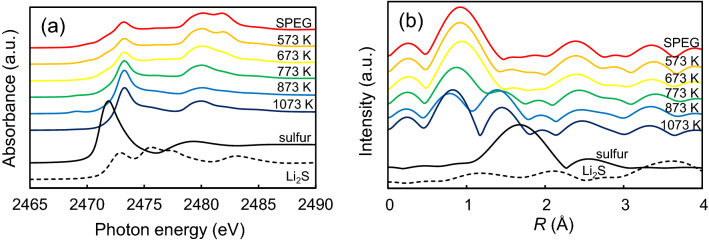
Local structures of SPEG after heat treatment at different temperatures and first discharge: (**a**) S *K*-edge XANES spectra and (**b**) S *K*-edge EXAFS spectra.

After the first discharge of lithiation, the cells were disassembled under an argon atmosphere inside the glove box and S *K*-edge XAFS analysis of the electrodes containing SPEG was conducted after the heat treatment. Figure 8a shows the S *K*-edge XANES spectra of lithiated SPEG electrodes. After lithiation, the XANES profile of the SPEG electrode differs from that of Li_2_S. An increase in temperature during the heat treatment leads to a sharper peak at 2473 eV. Similar to the XANES spectra, the S *K*-edge EXAFS spectra of lithiated SPEG electrodes (Fig. 8b) are different from those of Li_2_S. The spectra after the 873 and 1073 K treatment show a peak at 1.4 Å, which does not contribute to the electrochemical performance. Focusing on the spectra of SPEG before and after the heat treatments at 573 K and 673 K (Fig. 8b), the peak at 1.8 Å (Fig. 5b) was observed to weaken after lithiation; this peak represents the site where lithium is accepted in the SPEG matrix. A similar peak is found in rubeanic acid; thus, this peak can be assigned to the C=S bond. However, Li_2_S shows no peak at 1.8 Å, which indicates that sulfur in SPEG is not present as a molecule (like S_8_) but is chemically bound to carbon.

## Conclusions

In this study, the local structure and electrochemical performance of SPEG were investigated after heat treatment at various temperatures. Based on the correlation between the physicochemical properties and discharge/charge performances, it was concluded that the S–S and C=S bonds led to an electrochemical reaction in SPEG. This result indicated that the cooperative action of carbon and sulfur contributes to the electrochemical performance of SPEG. Further, the heat treatment of SPEG rearranges the carbon and sulfur in the SPEG matrix, while causing desulfurization. Currently, the reason for the failure of sulfur as an active material is not clear. A possible reason is that sulfur is buried deep inside a carbon matrix, thereby rendering it inaccessible for the electrolyte. Although the analysis of the local structure of SPEG is still being conducted, several important findings have been obtained using numerous spectroscopic techniques. We hope that our findings contribute to the development of a new positive electrode of sulfur for utilization in Li–S batteries, owing to its advantages such as low cost, environmental friendliness, high safety, high capacity, and long lifetime.

## Methods

### General

Analytical-grade solvents and reagents were used in this study without further purification.

### SPEG synthesis

SPEG used in this study was prepared using the same procedure as mentioned in our previous report^14^. The calculated amount of polyethylene glycol (KISHIDA CHEMICAL Co., Ltd., Japan; M_n_ = 4000 g mol^−1^) and sulfur (Hosoi Chemical Industry Co., Ltd., Japan; 99.9%) was mixed and placed in an alumina crucible (90 mm*ϕ*, 700 mm length), which was set in a stainless steel vessel. The mixture was heat-treated at 573 K for 2 h under nitrogen flow. During this process, hydrogen sulfide (H_2_S) was generated along with the formation of SPEG; therefore, an appropriate ventilation or H_2_S-trapping system was required. After cooling to room temperature, the obtained material was pulverized in a mortar using a pestle and was heated again at 673 K under nitrogen flow for more than 3 h to remove the excess amount of sulfur. Subsequently, SPEG was obtained as a black powder along with the samples heat treated at 573, 673, 773, 873, and 1073 K for 1 h under nitrogen flow.

### Characterizations

Thermogravimetric analysis was performed using a DTG-60 (Shimadzu Corp., Japan). A scan was carried out from 323 to 1023 K at a heating rate of 20 K min^−1^ in an argon atmosphere using an alumina pan. The true density of the samples was measured using the AccuPyc II 1340 pycnometer (Micromeritics Instrument Corp., U.S.A.), wherein helium gas was used as the displacement medium. The specific surface areas of the samples were determined using the Brunauer–Emmett–Teller (BET) method that utilized BELSORP-mini (BEL Japan, Inc.), and the particle size distribution was measured via the dry method using an SPR-7340 (NIKKISO Co., Ltd.)^21^.

### Spectroscopic analysis

The Raman spectra of the sample powders were analyzed with RAMANtouch VIS-NIR-LT (Nanophoton corp, Japan) by using a semiconductor laser to achieve an excitation with the wavelength of 532 nm. The Raman peak assigned to the silicon substrate at 520 cm^−1^ was used for the calibration.

High-energy X-ray total scattering experiments were carried out at the BL04B2 beam line using a two-axis diffractometer of the SPring-8 synchrotron radiation facility in Japan (Project No. 2017B1013). The photon energy (*E*) of the incident X-ray beam was 61.4 keV. The sample was sealed in a quartz capillary (0.3 mm diameter) with argon. The measurements were conducted in the transmission mode. The data were collected in the *Q* range from 0.2 to 25 Å^−1^ at room temperature, where *Q* is the magnitude of the scattering vector (*Q* = 4*πsinθ/λ*, *θ* is half of the scattering angle). The details of the correction and normalization procedures as well as the structure factor *S*(*Q*) were derived according to the previous literature^22^. Sulfur and hard carbon were used as the references.

S *K*-edge XANES analyses of SPEG were performed at BL-13 in the SR Center facility of Ritsumeikan University. XANES spectra were obtained in the total electron yield (TEY) mode. Rubeanic acid and L-cystine were used as references.

### Preparation of electrodes and battery test

SPEG electrode sheets were prepared by mixing SPEG, acetylene black (as a conductive carbon), and polytetrafluoroethylene in a mortar to achieve a weight ratio of 50:45:5. The sheet was then pressed onto a mesh-type aluminum current collector (20 μm thickness) and was dried in a dry chamber at room temperature for 24 h.

CR2032 coin-type cells were assembled using a lithium metal foil (Honjo Metal Co., Ltd., Japan) as the counter electrode, a microporous polypropylene membrane as the separator, and a 1:1 (in mole) mixture of lithium bis(trifluoromethylsulfonyl)amide (Solvay Japan, Ltd.) dissolved in tetraglyme (supplied from Nippon Nyukazai Co., Ltd., Japan) as the electrolyte. After adding a sufficient amount of electrolyte, the cell case was sealed. All procedures were carried out in a glove box filled with argon gas where the dew point was lower than 193 K.

The cells were galvanostatically discharged at a current density of 15 mA per gram of SPEG with a cut-off potential of 1.0 V (vs. Li^+^/Li) and were galvanostatically charged at the same current density with a cut-off potential of 3.0 V (vs. Li^+^/Li). The charge/discharge test was performed using a computer-controlled system (ABE System, Electro Field Co., Ltd., Japan) equipped with a thermostatic chamber at 303 K. In this study, the obtained capacities are expressed as the per unit mass of SPEG in the electrode^23^.

## Supplementary information

Supplementary Figures.
