# Supplementary material for: Local structure and electrochemical performances of sulfurized polyethylene glycol after heat treatment
Source: Sci Rep. 2020 Oct 9;10:16918. doi: 10.1038/s41598-020-74118-5 (PMC7547116; doi:10.1038/s41598-020-74118-5)
Supplement: Supplementary file 1 — Supplementary Figures. [file 41598_2020_74118_MOESM1_ESM.pdf]

Supplementary Information

for

Local Structure and Electrochemical Performances of Sulfurized Polyethylene Glycol after Heat Treatment

Nobuhiko Takeichi, Toshikatsu Kojima, Hiroshi Senoh, and Hisanori Ando*

Research Institute of Electrochemical Energy (RIECEN), National Institute of Advanced Industrial Science and Technology (AIST), 1-8-31 Midorigaoka, Ikeda, Osaka 563-8577, Japan.

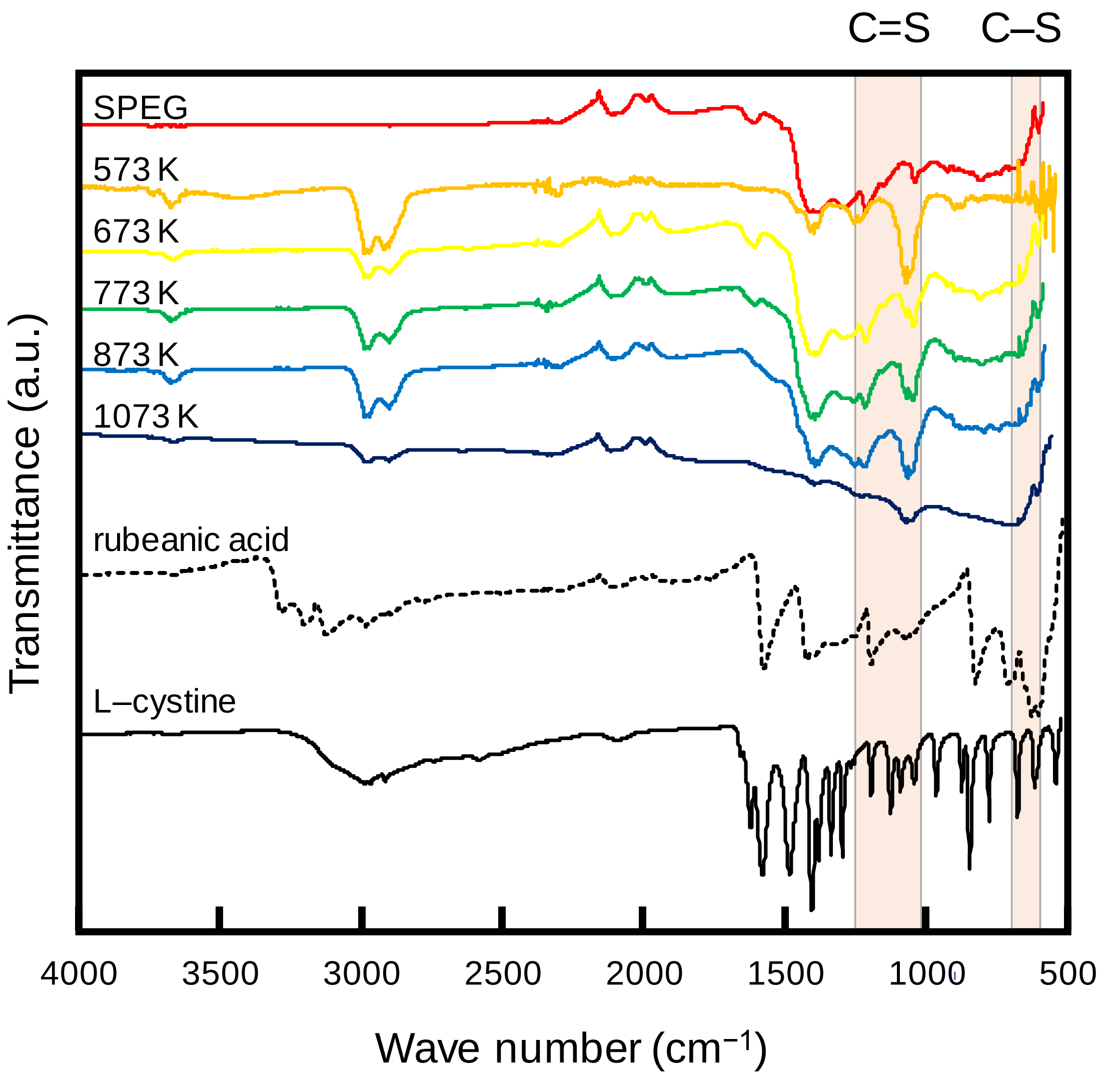

Figure S1. FT-IR spectra of SPEGs and standard compounds.

Methods:

FT-IR measurements were performed with Frontier™ MIR spectrometer (PerkinElmer, Inc.) using KBr window. The characteristic regions for C–S and C=S bonds are cited from the literature [S1].

[S1] R.M. Silverstein, G. Clayton Bassler, and Terence C. Morrill, "Spectrometric identification of organic compounds (fifth edition)", John Wiley & Sons, Inc., 1991.

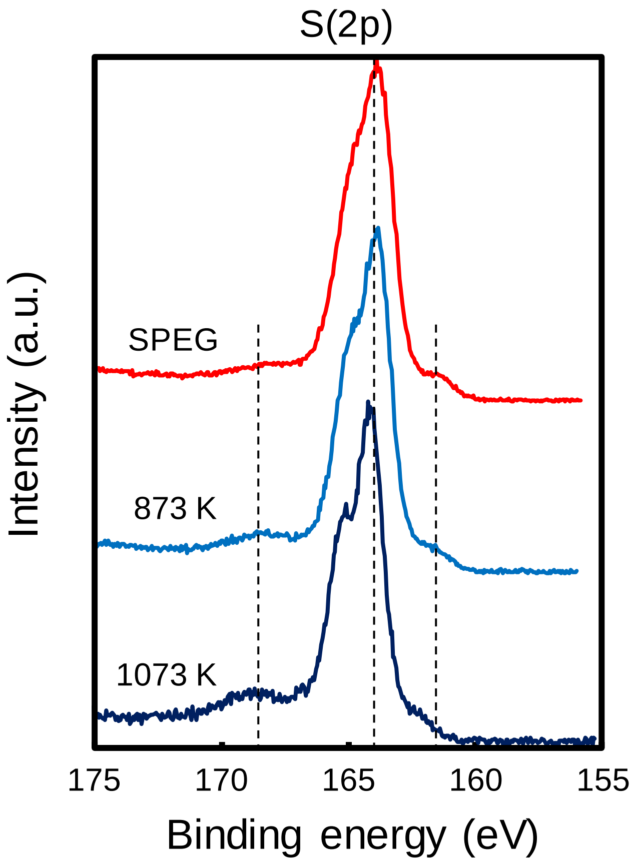

Figure S2. X-ray photoelectron spectra of SPEGs for S(2p).

Methods:

XPS measurements were performed with VersaProbe III (ULVAC-PHI, Inc.) using monochromatized Al-Kα radiation (hν=1486.6 eV). The sample was sputtered by argon ion (Ar-GCIB, 15 kV) neutralized with electron beam (1.0 V, 20 μA) prior to the measurement.

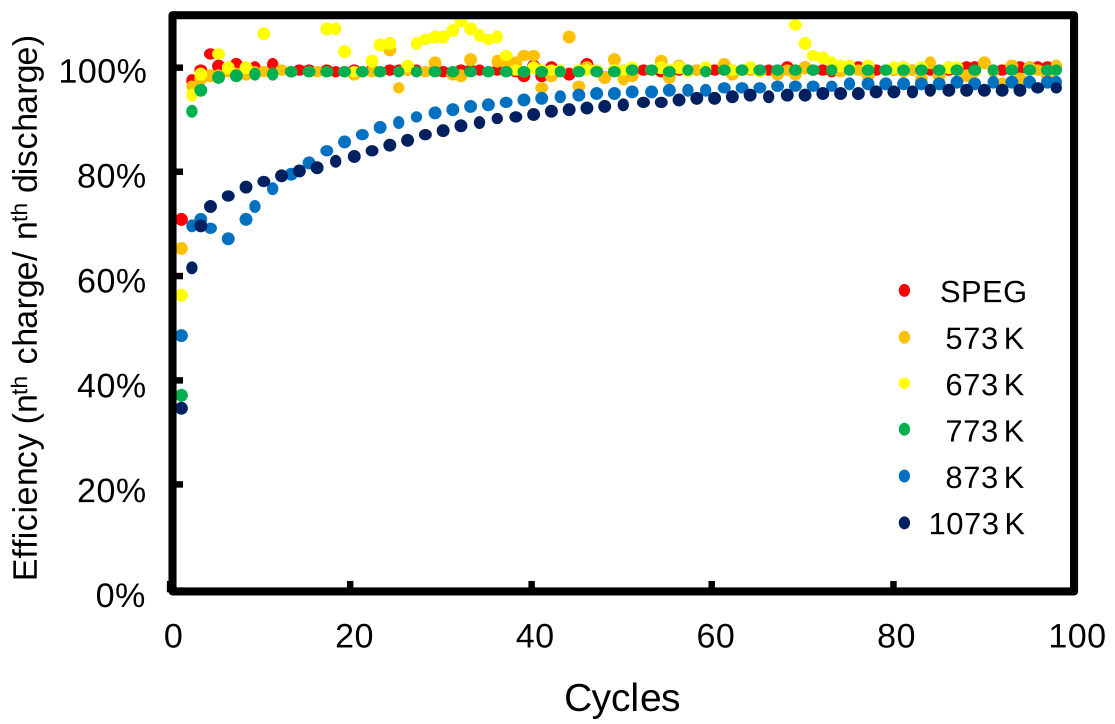

Figure S3. Coulombic efficiency of SPEG batteries treated at different temperatures. See 'Methods' section for the detail.
